# Rate of Responders for Post-Exercise Hypotension after Beach Tennis, Aerobic, Resistance and Combined Exercise Sessions in Adults with Hypertension

**DOI:** 10.3390/sports11030058

**Published:** 2023-03-06

**Authors:** Leandro de Oliveira Carpes, Lucas Betti Domingues, Sandra Costa Fuchs, Rodrigo Ferrari

**Affiliations:** 1 Graduate Program in Cardiology, School of Medicine, Universidade Federal do Rio Grande do Sul, Porto Alegre 90035-903, Brazil; 2 Sports and Exercise Training Study Group, Hospital de Clínicas de Porto Alegre, Porto Alegre 90035-903, Brazil; 3 Postgraduate Program in Human Movement Sciences, School of Physical Education, Universidade Federal do Rio Grande do Sul, Porto Alegre 90690-200, Brazil

**Keywords:** endurance exercise, strength exercise, high blood pressure, concurrent training, recreational sports

## Abstract

Post-exercise hypotension (PEH) is typically reported as mean values, but a great inter-individual variation in blood pressure (BP) response after a single exercise session is expected, especially when comparing different modalities of exercise. The purpose was to evaluate the inter-individual BP responses after beach tennis, aerobic, resistance and combined exercise sessions in adults with hypertension. We conducted a post hoc analysis of pooled crossover randomized clinical trials from six previously published studies of our research group, and analyzed data from 154 participants with hypertension (≥35 years). BP was assessed using office BP, and the mean changes throughout the 60 min after recreational beach tennis (BT, n = 23), aerobic (AE, n = 18), combined (COMB, n = 18), and resistance (RES, n = 95) exercise sessions were compared to a non-exercising control session (C). To categorize the participants as responders and non-responders for PEH, the typical error (TE) was calculated as follows: TE = SD_difference_/√2, where SD_difference_ is the standard deviation of the differences in BP measured before the interventions in the exercise and control sessions. Participants who presented PEH greater than TE were classified as responders. The TE was 7 and 6 mmHg for baseline systolic and diastolic BP, respectively. The rate of responders for systolic BP was as follows: BT: 87%; AE: 61%; COMB: 56%; and RES: 43%. For diastolic BP, the rate of responders was as follows: BT: 61%; AE: 28%; COMB: 44%; and RES: 40%. Results evidenced that there was a high inter-individual variation of BP after a single bout of different physical activity modalities in adults with hypertension, suggesting that exercise protocols with aerobic characteristics (i.e., BT, AE, and COMB sessions) presented PEH in most of its practitioners.

## 1. Introduction

The negative impact of sustained high blood pressure (BP) on cardiovascular morbidity and mortality [1] can be opposed with lifestyle changes aligned to the antihypertensive treatment [1]. Traditional physical exercises such as aerobic and resistance training are recognized as cornerstone non-pharmacological therapies in the prevention and management of hypertension [1,2,3]. Recently, recreational sports, with the purpose of participation, enjoyment, and social involvement, also proved to be effective to BP control [4]. Despite being widely recommended and having robust evidence on cardiovascular health [2], adherence to traditional exercises seems to be low [5]. Recreational sports have the advantage of being practiced in easily accessible facilities and public areas [6]. In particular, beach tennis, a racket sport derived from tennis and beach volleyball, emerges as a new option for these goals. The modality is played in doubles (2 vs. 2) on a sand court with similar dimensions of beach volleyball. A net divides the court in half at 1.70 cm in height, and slower balls than traditional tennis are used to play. Similar to traditional tennis, the players hit the ball every time it crosses the net, resulting in a great cardiovascular and neuromuscular demand during the match.

Acute BP reductions that occur following a single bout of exercise, a phenomenon termed post-exercise hypotension, may predict the extent of BP lowering following chronic training interventions [7]. Several studies have demonstrated this physiological phenomenon, and the intensity/volume/modality of exercise seems to influence the magnitude and duration of BP reduction after an exercise session [8,9,10]. Findings from systematic reviews and metanalyses suggest a reduction in systolic/diastolic BP after aerobic (≈6/4 mmHg) [8], combined (≈7/3 mmHg) [8] and resistance exercises (≈3/3 mmHg) [10] after 1 h when compared to the control session. Despite the growth of evidence on post-exercise hypotension, the effect of inter-individual acute BP response after exercise is scarcely evaluated. The response to the exercise at the group level represents the average effect and may not fully apply to all group members [11], as participants may reduce (responders), not change, or even increase BP (non-responders) after exercise [12,13,14].

Since adults with hypertension may have high inter-individual BP variability after different exercise modalities, and the lack of evidence on BP responders after a single bout of exercise, the aim of the present study was to evaluate the inter-individual BP responses after beach tennis, aerobic, resistance and combined exercise sessions in adults with hypertension.

## 2. Materials and Methods

### 2.1. Study Design and Participants

We conducted a post hoc analysis of pooled crossover randomized clinical trials (RCTs) from six previously published studies of our research group [9,15,16,17,18,19]. In two studies [9,16], participants performed two exercise protocols and one non-exercising control session. In these cases, data from both exercise protocols were considered in the analysis. All primary studies evaluated acute BP responses measured throughout the 60 min after exercise and non-exercising control sessions. Participants were adults (≥35 years) with a previous medical diagnosis of hypertension who had not practiced structured exercise in the three months prior to the beginning of the trials. Similar exclusion criteria were adopted in the original studies, including previous diagnosis of heart failure, current smoking, musculoskeletal problems that restrained subjects from exercising, and changes in antihypertensive medications throughout the trial. 

The WinPepi software calculator was used to estimate the sample size in the primary studies. The randomization of participants was generated using computer software by an epidemiologist that did not participate in the recruitment of participants or their assignment to the experimental sessions. The participants and the research team were blinded to the randomization list until the day in which the first experimental session was performed.

### 2.2. Characteristics of the Experimental Sessions

In the original trials, the non-exercising control and the exercise sessions were performed at the same time of the day to minimize diurnal variation in BP and residual effects of BP-lowering medications. Participants underwent two standardized BP measurements [20] on both arms and the arm with the highest value was used in pre- and post-intervention assessment. Participants were instructed to avoid any additional physical exercise, alcohol and stimulant substances throughout the studies, and not drink water during the experimental sessions. They were also asked to maintain their usual diet. Those who were taking BP-lowering medications were requested to maintain their current treatment throughout the studies.

The sequence of procedures started with the participants remaining at rest for 20 min to perform baseline office BP measurements, the implementation of the exercise or control protocol, and post-intervention BP measurements at 15 min intervals, during 60 min, using automatic oscillometric devices (Omron Hem, IL, USA [9,15,17,18] or Dinamap SX/P; Critikon, FL, USA [16,19]). The mean change in total 1 h on BP after beach tennis (n = 23), aerobic (n = 18), combined (n = 18), and resistance (n = 95) exercise sessions, and the corresponding non-exercising control sessions were assessed in the original trials. During the non-exercising control sessions, the participants remained at seated rest, and no exercise or the use of electronic devices (i.e., smartphones, notebooks) was allowed. 

#### 2.2.1. Beach Tennis 

Ref. [15] after a 5 min warm-up consisting of basic techniques, 3 beach tennis matches of 12 min with 2 min intervals between them were played. The regular beach tennis rules and a regular beach tennis court were used during the protocol. The 45 min of beach tennis was performed at an average intensity corresponding to 60–65% maximal oxygen consumption (VO_2max_).

#### 2.2.2. Aerobic Exercise 

Ref. [16] the aerobic protocol on a treadmill lasted 45 min and was performed at an intensity corresponding to 65–70% VO_2max_. In order to determine VO_2max_ and maximal heart rate, an incremental exercise test on treadmill was performed. The protocol consisted of an initial velocity of 3.5 km/h with 1% inclination during the first 2 min. Thereafter, velocity and grade were incremented by 0.4–0.6 km/h and 0.5–1.0% inclination, respectively, every 1 min until the participants reached their volitional exhaustion. The expired gas was analyzed using a metabolic cart (Oxycon Delta, VIASYS, Healthcare GmbH, Jaeger, Germany). The session was continuously monitored through the reserve heart rate to ensure that exercise intensity was maintained. Borg rating of perceived exertion equivalent (i.e., Borg scale 11–13) was used to the intensity control for patients taking beta-blockers.

#### 2.2.3. Combined Exercise 

Ref. [16] the combined aerobic and resistance exercise protocol lasted 45 min (20 min of resistance exercises plus 25 min of aerobic exercise on a treadmill). The aerobic exercise was performed at an intensity corresponding at 65–70% VO_2max_. During the resistance exercise protocol, 4 sets of 8 repetitions of 4 exercises were adopted: bench press, bilateral knee extensors, bilateral elbow flexors, and bilateral knee flexors. An active interval of two min was allowed between sets for each exercise (i.e., exercises were grouped in a block of two, and within each block, the sets of the second exercise were performed during the rest of the first). Each contraction (concentric and eccentric) lasted 1.5 s and was controlled by an electronic metronome. The prescription of the resistance exercise intensity was based on a percentage of one repetition maximum test (1RM) performed in the four exercises adopted during the experimental session: bench press and bilateral elbow flexors (free weight), bilateral knee extensors, and bilateral knee flexors (machines). Briefly, participants warmed up for 5 min on a cycle ergometer, performed light and brief stretching for all major muscle groups, and practiced specific movements with 1 set of 15 repetitions with light load in each exercise evaluated (30–40% of the estimated 1RM test load). Each subject’s maximal load (i.e., 1RM) was determined with no more than five attempts with a five-minute recovery period between sets. The resistance exercises during the combined training session were performed at 70%1RM.

#### 2.2.4. Resistance Exercise 

*Resistance exercise* data resulted from 4 studies with different protocols, as follows: 

Ref. [9], during the two resistance exercise sessions, different intensities were adopted (i.e., 50% and 75%1RM). The participants performed 4 sets of 10 repetitions in five exercises: bench press, knee extension, front pulldown, and knee flexion. A 2 min interval between sets and exercises was adopted. 

Ref. [17], after a warm-up set of 5 repetitions, 3 sets of 30 s were performed in 4 exercises: inverted row using a bar, squat, push-up, and sit-up. The exercise intensity was based on body-weight only, and participants were instructed to perform the maximum number of repetitions during each set. Each repetition was executed using volitional velocity. A 2 min interval between sets and exercises was adopted.

Ref. [18], three sets of 8 repetitions at 50%1RM in six exercises: seated row, knee extension, peck deck, knee flexion, elbow flexion, and elbow extension. The concentric phase of each repetition was performed “as fast as possible” and the eccentric phase lasted 1–2 s in duration. A 2 min interval between sets and exercises was adopted. 

Ref. [19], three sets of 10 repetitions at 50%1RM in five exercises: leg press, bench press, knee extension, upright row, and knee flexion. The concentric phase of each repetition was performed “as fast as possible” and the eccentric phase lasted 1–2 s in duration. A 2 min interval between sets and exercises was adopted. 

### 2.3. Classification of Responders and Non-Responders

In order to assess the inter-individual variability of PEH in each trial, the net-effect of each exercise session was calculated by the difference between BP responses observed in the exercise and the control sessions, as follows: PEH net-effect = [(post-exercise BP − baseline BP in the exercise session) − (post-control BP − baseline BP in the control session)]. To categorize the participants as responders and non-responders, the typical error was calculated, as follows: typical error = SD_difference_/√2, where SD_difference_ is the standard deviation of the differences in BP measured before the interventions in the exercise and control sessions. Then, participants who presented PEH greater than typical error were classified as responders.

### 2.4. Statistical Analyses 

Results were expressed as mean and standard deviation or standard error for variables with normal distribution. The assumption of normality and homogeneity parameters were checked with Shapiro–Wilk and Levene tests, respectively. Responders and non-responders were compared for clinical characteristics using t-test for independent samples and a chi-square or Fisher’s exact test to verify the association with the use of antihypertensive medication. 

Effect sizes were calculated using the following formula: (Mean of responders − Mean of non-responders)/pooled SD. The interpretation of the effect size adopted were based on the following criteria: <0.50, small; 0.50 to 0.79, medium; and ≥0.80, large. 

Statistical significance was set at *p* < 0.05. All statistical analyses were performed using SPSS Statistics for Windows version 22.0 (IBM, Armonk, NY, USA).

## 3. Results

The participants (n = 154/men = 62%) were aged 56 ± 12 years and had body mass index 29 ± 5 kg/m^2^. Thirty-four participants (22%) were not taking antihypertensive medications, 47 (31%) were taking one, 61 (40%) were taking two, and 12 (7%) were taking three antihypertensive medications. The classes of antihypertensive drugs were: diuretics: n = 64 (42%); β blockers: n = 35 (23%); angiotensin converting enzyme inhibitor: n = 32 (21%); angiotensin receptor antagonists: n = 19 (12%); calcium channel blockers: n = 25 (16%); and angiotensin II receptor blockers: n = 35 (23%). Approximately 60% of the sample had controlled BP (127 ± 13 mmHg systolic BP and 76 ± 10 mmHg diastolic BP).

**Figure 1:**
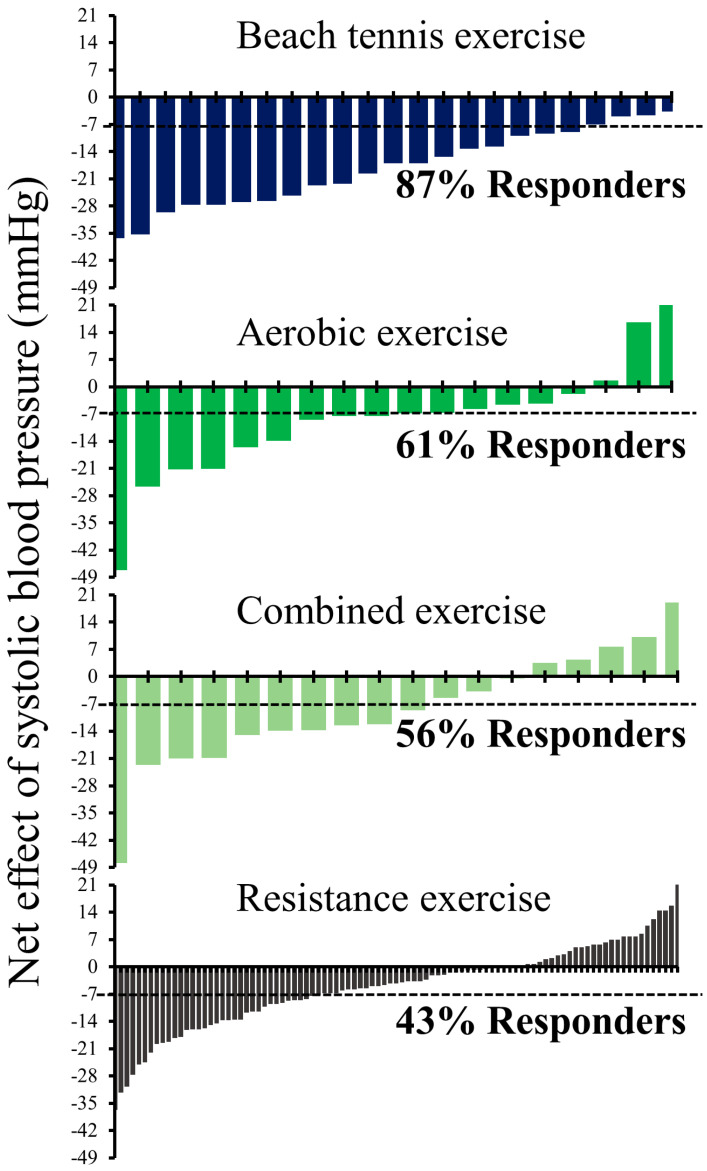
Individual changes in systolic blood pressure (exercise session minus control session). Dashed line: minimal detectable change (7 mmHg).

**Figure 2:**
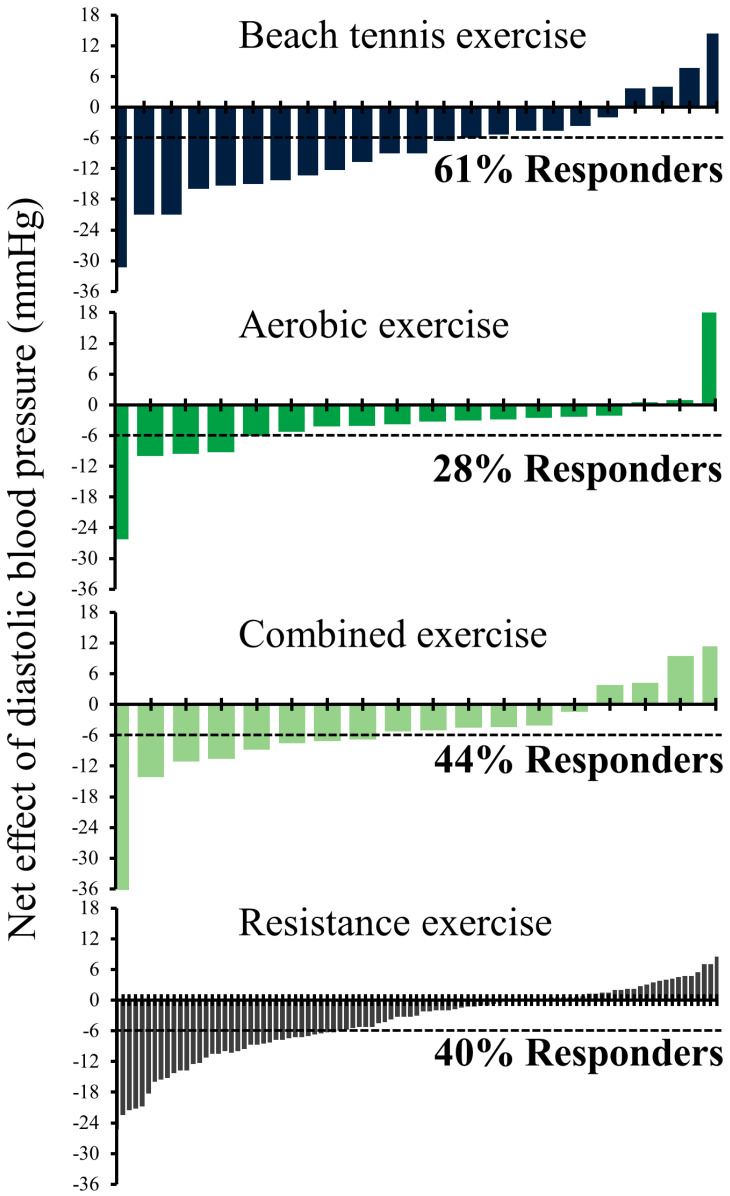
Individual changes in diastolic blood pressure (exercise session minus control session). Dashed line: minimal detectable change (6 mmHg).

The typical error of baseline BP (7 and 6 mmHg for systolic and diastolic BP, respectively) was used for the analyses of responders. The rate of responders and non-responders after different modalities of exercise are shown in Figure 1 and Figure 2. For systolic BP, responders were: beach tennis: 87%, aerobic: 61%, combined: 56%, and resistance: 43%, while for diastolic BP, the percentage of responders dropped to: 61%, 44%, 40%, and 28%, respectively.

Table 1 and Table 2 present the blood pressure characteristics of responders and non-responders according to the exercise modality, and the corresponding effect size.

Table 3 describes the characteristics of anti-hypertensive medications for responders and non-responders of the beach tennis, aerobic, combined, and resistance exercises. No statistically significant differences regarding the use of antihypertensive medication of responders and non-responders were found (*p* > 0.05).

## 4. Discussion

We performed this exploratory analysis to classify the rate of responders for post-exercise hypotension after beach tennis, aerobic, combined, and resistance exercises modalities. To the best of our knowledge, no previous studies have compared BP-lowering effects of recreational sport and traditional exercise modalities and evaluated inter-individual variation of BP between them in adults with hypertension. The main result showed difference in the rate of responders among the different exercise modalities and suggested that not all participants present post-exercise hypotension response during the first hour after exercise. Beach tennis showed 87% of responders for systolic BP, followed by aerobic (61%), combined (56%), and resistance (42%). These findings are partially in accordance with the physical activity guidelines for adults [21] and for the treatment and management of hypertension [22], which recommend aerobic exercise as a first line of non-pharmacological treatment, followed by strength exercises to complete a physical training program.

Physical activities with a higher cardiovascular demand during its practice seem to be the best option to induce post-exercise hypotension, since some physiological responses related to this type of exercise (i.e., decreased alpha-adrenergic response to sympathetic stimuli) reduce peripheral vascular resistance, and others (i.e., the secretion of vasodilation substances) help to maintain the vasodilation after exercise [23,24,25,26]. Based on that, we could expect a higher rate of responders after aerobic and beach tennis exercises, as observed in the average effect of the original studies [15,16]. Differences on the participants’ characteristics of these two trials such as age, sex, and baseline blood pressure could have influenced the magnitude of post-exercise hypotension. We also speculate that the greater hypotensive effect after beach tennis may be related to the intermittent characteristic of the game, allowing the individuals to reach higher intensities than continuous aerobic exercise, even though the sessions were time-equivalent (45 min) and the overall intensity of the sessions were similar (60–70%VO_2max_). Future studies directly comparing aerobic and beach tennis protocols and the post-exercise hypotension mechanisms are encouraged to better understand the difference on the rate of responders after these modalities of exercise.

Few studies have evaluated the rate of responders for post-exercise hypotension, with statistical methods and BP values different from each other. In the study of Costa et al., (2016) [27], healthy normotensive men were evaluated (n = 14; ≈24 years) for BP throughout 60 min after high-intensity interval exercise and continuous exercise, and the minimal detectable change for systolic/diastolic BP to define the responders was 5.8/7.0 mmHg. The rate of responders for high-intensity interval exercise was 36%/7% and 64%/21% after continuous exercise, for systolic and diastolic BP, respectively. In another study that pooled data from seven previous trials (n = 131; ≈36 years) on post-exercise hypotension after resistance exercise protocols [28], participants who presented a reduction greater than the typical error of baseline (systolic BP 3.9 mmHg and diastolic BP 4.2 mmHg) were classified as responders. In this trial, even evaluating post-exercise blood pressure with intervals of 15 min (e.g., 45, 60 and 90 min after the sessions), only the moment of greatest BP reduction was considered for data analysis, resulting in 69% being responders for systolic BP and 45% for diastolic BP. It suggests that different methods adopted to calculate the rate of responders can influence the results and confirm that the same exercise protocol serves as an antihypertensive lifestyle therapy for some, but not all.

The unique finding of this trial is that beach tennis had the highest rate of responders. This modality of recreational sport allows people of different ages and technical and physical levels to play and requires only 2–4 participants per match. These characteristics make this modality quite democratic, allowing practitioners to be able to practice in a few sessions of familiarization with the sport. Moreover, the soft sandy floor provides a pleasant feeling, and its instability requires more muscle work to move with greater energy expenditure [29]. Based on these advantages, associated with its efficacy to acutely reduce BP [15], it could be used as a valuable tool of the non-pharmacological treatment for adults with hypertension. Additional benefits on cardiovascular health, cardiorespiratory fitness, and body composition with relatively low perceived exertion rates [4,15,30,31] reinforce the importance of recreational sports in this population.

Some limitations of the present study should be considered when interpreting the results. The original trials included untrained participants at a specific age group (men and women between 35 to 75 years old), limiting the generalizability of results to other populations. Even so, as an original exploratory analysis, the results allowed the broadening of the view on exercise protocols. In addition, the novelty is the beach tennis, which has only one protocol of exercise tested, besides aerobic and combined exercise, while resistance exercise has five protocols included. The strengths of this study also include the use of data of six RCTs that adopted a similar standardized methodology, and the use of an oscillometric BP measurement device to assess the outcome, a method that is not dependent on the appraiser. Additionally, to explore data from participants with similar characteristics (e.g., adults with hypertension, physically inactive, living in the same city) is a plus. Finally, our sample was mostly composed of individuals with well-controlled hypertension and using anti-hypertensive medications. We could expect a greater rate of responders in those with higher resting BP values or not taking anti-hypertensive medications. The training status and sex of the participants may also contribute to the rate of responders for post-exercise hypotension and should be taken into account in future investigations on this topic.

## 5. Conclusions

In summary, a high inter-individual variation of BP was found after beach tennis and traditional exercise modalities, suggesting that exercise protocols with aerobic characteristics (i.e., beach tennis and aerobic exercise sessions) result in the highest rate of responders for post-exercise hypotension. For the non-responders, personalized exercise interventions with appropriate stimuli (i.e., volume, intensity) may be effective in lowering BP but require further investigation.

## Figures and Tables

**Table 1:** Differences in characteristics between responders (>7 mmHg for systolic BP) and non-responders in each exercise modality.

		Responder	Non-Responder	Δ	Effect Size	*p* Value
**Aerobic exercise**		11 (61)	7 (39)			
Systolic BP, mmHg						
	Pre Exercise	123 ± 10 (117 to 130)	114 ± 6 (109 to 120)	9 ± 4 (1 to 18)	1.03	**0.042**
	Mean post 1 h Exercise	115 ± 7 (111 to 120)	113 ± 10 (104 to 121)	2 ± 4 (−6 to 11)	0.24	0.524
	Pre Control	120 ± 10 (113 to 126)	123 ± 16 (108 to 138)	−3 ± 6 (−16 to 10)	0.24	0.601
	Mean post 1 h Control	127 ± 14 (119 to 137)	117 ± 12 (106 to 129)	10 ± 6 (−3 to 24)	0.75	0.116
	Net effect	−17 ± 12 (−25 to −8)	4 ± 12 (−7 to 15)	−21 ± 6 (−33 to −8)	1.75	**0.003**
Diastolic BP, mmHg						
	Pre Exercise	75 ± 12 (67 to 83)	68 ± 7 (62 to 75)	7 ± 5 (−4 to 17)	0.67	0.208
	Mean post 1 h Exercise	71 ± 9 (65 to 77)	70 ± 8 (62 to 77)	1 ± 4 (−7 to 10)	0.12	0.696
	Pre Control	74 ± 9 (68 to 80)	68 ± 13 (57 to 80)	6 ± 5 (−5 to 16)	0.56	0.271
	Mean post 1 h Control	77 ± 10 (70 to 83)	70 ± 9 (62 to 78)	7 ± 5 (−2 to 17)	0.73	0.134
	Net effect	−7 ± 7 (−11 to −2)	0 ± 10 (−9 to 9)	−7 ± 4 (−15 to 2)	0.85	0.129
**Combined exercise**		10 (56)	8 (44)			
Systolic BP, mmHg						
	Pre Exercise	128 ± 7 (122 to 133)	118 ± 10 (109 to 126)	10 ± 4 (1 to 19)	1.18	**0.027**
	Mean post 1 h Exercise	117 ± 5 (111 to 124)	116 ± 10 (108 to 125)	1 ± 5 (−9 to 11)	0.13	0.800
	Pre Control	120 ± 10 (111 to 127)	123 ± 15 (110 to 135)	−3 ± 6 (−15 to 10)	0.24	0.631
	Mean post 1 h Control	129 ± 14 (117 to 139)	117 ± 11 (108 to 126)	12 ± 6 (1 to 25)	0.94	**0.049**
	Net effect	−19 ± 11 (−28 to −11)	4 ± 8 (−2 to 11)	−23 ± 5 (−33 to −13)	2.35	**<0.001**
Diastolic BP, mmHg						
	Pre Exercise	76 ± 11 (67 to 85)	71 ± 7 (65 to 77)	5 ± 5 (−5 to 15)	0.63	0.324
	Mean post 1 h Exercise	71 ± 5 (70 to 74)	70 ± 9 (62 to 77)	1 ± 3 (−6 to 8)	0.14	0.789
	Pre Control	73 ± 10 (64 to 80)	71 ± 11 (61 to 80)	2 ± 5 (−9 to 13)	0.19	0.712
	Mean post 1 h Control	77 ± 10 (69 to 84)	70 ± 9 (63 to 78)	7 ± 4 (−3 to 16)	0.73	0.142
	Net effect	−9 ± 11 (−18 to −1)	−1 ± 8 (−8 to 6)	−8 ± 5 (−18 to 1)	0.82	0.093
**Resistance exercise**		41 (43)	54 (57)			
Systolic BP, mmHg						
	Pre Exercise	128 ± 14 (124 to 132)	127 ± 14 (123 to 131)	1 ± 3 (−5 to 6)	0.07	0.792
	Mean post 1 h Exercise	119 ± 24 (111 to 127)	129 ± 13 (125 to 132)	−10 ± 4 (−17 to −2)	0.54	**0.018**
	Pre Control	126 ± 13 (122 to 130)	132 ± 13 (128 to 135)	−6 ± 3 (−11 to −1)	0.46	**0.025**
	Mean post 1 h Control	135 ± 17 (129 to 140)	132 ± 14 (128 to 136)	3 ± 3 (−3 to 3)	0.20	0.351
	Net effect	−14 ± 9 (−17 to −12)	2 ± 7 (0 to 4)	−16 ± 2 (−19 to −12)	2.02	**<0.001**
Diastolic BP, mmHg						
	Pre Exercise	77 ± 11 (74 to 80)	75 ± 9 (72 to 77)	2 ± 2 (−2 to 7)	0.20	0.231
	Mean post 1 h Exercise	71 ± 15 (66 to 76)	76 ± 8 (74 to 78)	−5 ± 2 (−10 to −1)	0.43	**0.032**
	Pre Control	76 ± 11 (73 to 79)	77 ± 9 (74 to 79)	−1 ± 2 (−5 to 3)	0.10	0.716
	Mean post 1 h Control	80 ± 10 (77 to 83)	79 ± 9 (77 to 82)	1 ± 2 (−3 to 5)	0.11	0.741
	Net effect	−8 ± 8 (−11 to −6)	−1 ± 5 (−3 to 0)	−7 ± 1 (−9 to −4)	1.08	**<0.001**
**Beach tennis exercise**		20 (87)	3 (13)			
Systolic BP, mmHg						
	Pre Exercise	129 ± 12 (124 to 134)	127 ± 6 (113 to 140)	2 ± 7 (−12 to 17)	0.17	0.740
	Mean post 1 h Exercise	112 ± 12 (107 to 118)	117 ± 2 (112 to 122)	−5 ± 7 (−19 to 10)	0.44	0.491
	Pre Control	126 ± 13 (120 to 132)	128 ± 14 (91 to 164)	−2 ± 8 (−19 to 16)	0.15	0.842
	Mean post 1 h Control	130 ± 12 (124 to 135)	122 ± 10 (97 to 147)	8 ± 7 (−8 to 23)	0.68	0.349
	Net effect	−21 ± 9 (−25 to −16)	−5 ± 1 (−6 to −3)	−16 ± 2 (−20 to −12)	1.87	**<0.001**
Diastolic BP, mmHg						
	Pre Exercise	82 ± 8 (78 to 86)	70 ± 4 (60 to 80)	12 ± 5 (2 to 22)	1.56	**0.025**
	Mean post 1 h Exercise	75 ± 8 (71 to 78)	70 ± 3 (63 to 80)	5 ± 5 (−5 to 14)	0.65	0.350
	Pre Control	80 ± 10 (75 to 85)	73 ± 5 (60 to 85)	7 ± 6 (−5 to 20)	0.73	0.249
	Mean post 1 h Control	82 ± 10 (78 to 87)	72 ± 9 (51 to 94)	10 ± 6 (−3 to 23)	1.01	0.111
	Net effect	−10 ± 9 (−14 to −5)	1 ± 5 (−12 to 14)	−11 ± 4 (−21 to −1)	1.26	0.038

Values are Means ± standard deviation or standard error for Δ values (95% Confidence Interval); Net effect = [(post-exercise BP − baseline BP in the exercise session) − (post-control BP − baseline BP in the control session)]; Bold *p*-values indicate significant results (*p* < 0.05).

**Table 2:** Differences in characteristics between responders (>6 mmHg for systolic BP) and non-responders in each exercise modality.

		Responder	Non-Responder	Δ	Effect Size	*p* Value
Aerobic exercise, n (%)			5 (28)	13 (72)		
Systolic BP, mmHg						
	Pre Exercise	126 ± 9 (115 to 137)	118 ± 9 (112 to 123)	8 ± 5 (−2 to 18)	0.89	0.102
	Mean post 1 h Exercise	116 ± 5 (109 to 122)	114 ± 9 (108 to 119)	2 ± 4 (−6 to 11)	0.24	0.609
	Pre Control	115 ± 7 (106 to 124)	123 ± 13 (115 to 131)	−8 ± 6 (−22 to 5)	0.68	0.214
	Mean post 1 h Control	125 ± 13 (109 to 141)	123 ± 14 (114 to 132)	2 ± 7 (−14 to 18)	0.15	0.767
	Net effect	−20 ± 17 (−41 to 0)	−4 ± 13 (−12 to 4)	−16 ± 7 (−32 to −1)	1.13	**0.047**
Diastolic BP, mmHg						
	Pre Exercise	79 ± 15 (61 to 97)	70 ± 8 (65 to 75)	9 ± 5 (−2 to 20)	0.88	0.116
	Mean post 1 h Exercise	73 ± 10 (61 to 84)	70 ± 8 (65 to 75)	3 ± 4 (−6 to 13)	0.35	0.499
	Pre Control	71 ± 13 (55 to 88)	72 ± 10 (66 to 78)	−1 ± 6 (−13 to 11)	0.09	0.896
	Mean post 1 h Control	78 ± 13 (62 to 94)	73 ± 8 (67 to 78)	5 ± 5 (−6 to 16)	0.11	0.316
	Net effect	−12 ± 8 (−22 to −2)	−1 ± 7 (−5 to 3)	−11 ± 4 (−19 to −4)	1.51	**0.007**
Combined exercise, n (%)			8 (44)	10 (56)		
Systolic BP, mmHg						
	Pre Exercise	124 ± 10 (115 to 132)	122 ± 10 (115 to 129)	2 ± 5 (−8 to 12)	0.20	0.700
	Mean post 1 h Exercise	115 ± 10 (106 to 123)	119 ± 9 (112 to 125)	−4 ± 4 (−14 to 5)	0.42	0.378
	Pre Control	121 ± 11 (111 to 130)	121 ± 14 (110 to 131)	0 ± 6 (−13 to 13)	0.00	0.908
	Mean post 1 h Control	124 ± 13 (114 to 135)	123 ± 15 (110 to 133)	1 ± 7 (−13 to 15)	0.07	0.887
	Net effect	−13 ± 19 (−28 to 3)	−5 ± 12 (−14 to 5)	−8 ± 7 (−23 to 7)	0.52	0.288
Diastolic BP, mmHg						
	Pre Exercise	76 ± 11 (67 to 85)	71 ± 8 (65 to 77)	5 ± 5 (−5 to 15)	0.53	0.308
	Mean post 1 h Exercise	68 ± 6 (64 to 73)	72 ± 8 (66 to 78)	−4 ± 3 (−10 to 4)	0.56	0.335
	Pre Control	71 ± 10 (62 to 79)	73 ± 11 (63 to 81)	−2 ± 5 (−13 to 8)	0.19	0.648
	Mean post 1 h Control	76 ± 10 (69 to 84)	73 ± 10 (65 to 78)	3 ± 5 (−7 to 13)	0.30	0.583
	Net effect	−13 ± 10 (−21 to −5)	0 ± 6 (−4 to 6)	−13 ± 4 (−21 to −5)	1.63	**0.003**
Resistance exercise, n (%)			38 (40)	57 (60)		
Systolic BP, mmHg						
	Pre Exercise	128 ± 13 (123 to 132)	127 ± 15 (124 to 131)	1 ± 3 (−6 to 6)	0.07	0.950
	Mean post 1 h Exercise	122 ± 17 (116 to 127)	127 ± 22 (121 to 132)	−5 ± 4 (−13 to 3)	0.25	0.255
	Pre Control	125 ± 12 (121 to 129)	132 ± 13 (128 to 135)	−7 ± 3 (−12 to −1)	0.56	**0.015**
	Mean post 1 h Control	131 ± 15 (126 to 136)	134 ± 15 (130 to 138)	−3 ± 3 (−10 to 3)	0.20	0.309
	Net effect	−12 ± 11 (−16 to −8)	−1 ± 8 (−3 to 2)	−11 ± 2 (−15 to −7)	1.18	<0.001
Diastolic BP, mmHg						
	Pre Exercise	78 ± 10 (75 to 81)	75 ± 10 (72 to 77)	3 ± 2 (−1 to 7)	0.30	0.103
	Mean post 1 h Exercise	72 ± 10 (69 to 75)	75 ± 13 (72 to 79)	−3 ± 3 (−8 to 2)	0.25	0.186
	Pre Control	74 ± 9 (71 to 77)	78 ± 10 (75 to 81)	−4 ± 2 (−8 to −1)	0.42	**0.036**
	Mean post 1 h Control	79 ± 10 (76 to 82)	80 ± 10 (77 to 83)	−1 ± 2 (−5 to 3)	0.10	0.656
	Net effect	−12 ± 5 (−13 to −10)	0 ± 3 (−1 to 1)	−12 ± 1 (−14 to −10)	3.06	**<0.001**
Beach tennis exercise, n (%)			14 (61)	9 (39)		
Systolic BP, mmHg						
	Pre Exercise	126 ± 12 (119 to 133)	133 ± 7 (127 to 139)	−7 ± 5 (−16 to 3)	0.67	0.145
	Mean post 1 h Exercise	108 ± 12 (102 to 115)	120 ± 5 (115 to 124)	−12 ± 4 (−20 to −2)	1.21	**0.014**
	Pre Control	127 ± 14 (119 to 135)	124 ± 12 (115 to 134)	3 ± 6 (−9 to 15)	0.23	0.592
	Mean post 1 h Control	132 ± 12 (125 to 139)	123 ± 11 (115 to 132)	9 ± 5 (−1 to 19)	0.77	0.088
	Net effect	−22 ± 9 (−27 to −17)	−12 ± 9 (−19 to −6)	−10 ± 4 (−18 to −2)	1.11	**0.015**
Diastolic BP, mmHg						
	Pre Exercise	83 ± 9 (76 to 88)	76 ± 7 (70 to 82)	7 ± 4 (−1 to 14)	0.84	0.075
	Mean post 1 h Exercise	72 ± 7 (68 to 76)	78 ± 7 (72 to 83)	−6 ± 3 (−12 to 0)	0.86	0.063
	Pre Control	81 ± 12 (75 to 88)	75 ± 4 (72 to 78)	6 ± 4 (−2 to 15)	0.61	0.121
	Mean post 1 h Control	85 ± 10 (79 to 91)	75 ± 8 (69 to 81)	10 ± 4 (2 to 18)	1.08	**0.022**
	Net effect	−14 ± 7 (−18 to −10)	1 ± 7 (−4 to 6)	−15 ± 3 (−21 to −9)	2.14	**<0.001**

Values are Means ± standard deviation or standard error for Δ values (95% Confidence Interval); Net effect = [(post-exercise BP − baseline BP in the exercise session) − (post-control BP − baseline BP in the control session)]; Bold *p*-values indicate significant results (*p* < 0.05).

**Table 3:** Characteristics of anti-hypertensive medications between responders (>7 mmHg for systolic blood pressure or >6 mmHg for diastolic blood pressure) and non-responders who did not present this response in each exercise modality.

		Aerobic Exercise		Combined Exercise		Resistance Exercise		Beach Tennis Exercise	
		Responder	Non-Responder	Responder	Non-Responder	Responder	Non-Responder	Responder	Non-Responder
**Systolic blood pressure**									
Anti-hypertensive medications, n (%)									
	Diuretics	5 (46)	1 (14)	3 (30)	3 (38)	22 (54)	22 (41)	8 (40)	1 (33)
	β blockers	4 (36)	1 (14)	4 (40)	1 (13)	6 (15)	14 (26)	4 (20)	1 (33)
	Angiotensin converting enzyme inibitors	3 (27)	2 (29)	3 (30)	2 (25)	5 (12)	11 (20)	5 (25)	1 (33)
	Angiotensin receptor antagonists	2 (18)	0 (0)	1 (10)	1 (13)	9 (22)	6 (11)	−	−
	Calcium channel blockers	4 (36)	3 (43)	3 (30)	4 (50)	3 (7)	5 (9)	2 (10)	1 (33)
	Angiotensin II receptor blockers	−	−	−	−	7 (17)	20 (37)	7 (35)	1 (33)
**Diastolic blood pressure**									
Anti-hypertensive medications, n (%)									
	Diuretics	2 (40)	4 (31)	1 (13)	5 (50)	19 (50)	25 (44)	4 (29)	5 (56)
	β blockers	1 (20)	4 (31)	2 (25)	3 (30)	7 (18)	13 (23)	3 (21)	2 (22)
	Angiotensin converting enzyme inibitors	2 (40)	3 (23)	2 (25)	3 (30)	5 (13)	11 (19)	4 (29)	2 (22)
	Angiotensin receptor antagonists	0 (0)	2 (15)	2 (25)	0 (0)	7 (18)	8 (14)	−	−
	Calcium channel blockers	2 (40)	5 (39)	3 (38)	4 (40)	4 (11)	4 (7)	2 (14)	1 (11)
	Angiotensin II receptor blockers	−	−	−	−	8 (21)	19 (33)	4 (29)	4 (44)

## Data Availability

The datasets generated during and/or analyzed during the current study are available from the corresponding author on reasonable request.
